# Subarachnoid space in infancy and early childhood: a systematic review of measurements

**DOI:** 10.1007/s00247-026-06640-z

**Published:** 2026-06-06

**Authors:** Jennifer Xu, Sofia Valencia Osorio, M. Katherine Henry, Susan Sotardi, Joanne N. Wood, Jakob Seidlitz, Aaron Alexander-Bloch, Jamie Catalano, Barbara H. Chaiyachati

**Affiliations:** 1 Lifespan Brain Institute, Children’s Hospital of Philadelphia and University of Pennsylvania, Philadelphia, USA; 2 Charles E. Schmidt College of Medicine, Florida Atlantic University, Boca Raton, USA; 3 PolicyLab, Children’s Hospital of Philadelphia, Philadelphia, USA; 4 Department of Pediatrics, Perelman School of Medicine, University of Pennsylvania, Division of General Pediatrics, 3500 Civic Center Blvd, HUB 15, Philadelphia, PA 19104 USA; 5 Clinical Futures, Children’s Hospital of Philadelphia, Philadelphia, USA; 6 Department of Radiology, Children’s Hospital of Philadelphia, Philadelphia, USA; 7 Department of Child and Adolescent Psychiatry and Behavioral Sciences, Children’s Hospital of Philadelphia, Philadelphia, USA

**Keywords:** Benign enlargement of the subarachnoid space, Macrocephaly, Subarachnoid space, Neuroimaging, Craniocortical width, Sinocortical width, Interhemispheric width

## Abstract

**Background:**

Benign enlargement of the subarachnoid space (BESS) is a condition characterized by widened subarachnoid spaces, typically associated with macrocephaly or increasing head circumference during infancy or early childhood. Often described as idiopathic and self-limiting, BESS has also been associated with developmental delays and spontaneous subdural collections. Yet, complete interrogation of these associations is limited by lack of normative subarachnoid space characterization and standardized diagnostic criteria.

**Objective:**

This project aimed to review and synthesize literature on imaging criteria and subarachnoid space measurements for BESS in infants and young children.

**Materials and methods:**

A systematic PubMed search was conducted using BESS-related terminology, informed by preliminary findings from a manual snowball review. We limited results to reports that provided subarachnoid space measurements from computed tomography (CT), magnetic resonance imaging (MRI), or ultrasound (US) neuroimaging and investigated subjects with or without BESS and/or macrocephaly from birth to 5 years old. Reports with concomitant neurological conditions affecting brain size were excluded.

**Results:**

The final collection included twenty-four reports (US *n*=10, CT *n*=6, MRI *n*=6, multiple *n*=2), consisting of 2,731 infants and children aged 0–36 months. Most reports evaluated non-BESS/macrocephaly subjects (*n*=15), followed by BESS and/or macrocephalic subjects (*n*=3) or both (*n*=6). Subarachnoid space measurements which were not expressly designated or were without detailed descriptions were the most commonly reported subarachnoid space index measure (*n*=14), followed by craniocortical width (CCW, *n*=9). Upper CCW measurements for non-BESS/macrocephaly subjects in US reports ranged from 5 mm to 10.86 mm. In general, for each study that included both non-BESS/macrocephaly and BESS and/or macrocephaly subjects, measurements reported were greater among BESS and/or macrocephaly subjects. There was some evidence of overlapping measurements between the subjects, such as those of CCW measurements in US studies.

**Conclusion:**

Overall, substantial variability was observed in imaging methodology and measurement thresholds. This review underscores the need for standardized diagnostic criteria to improve the consistency and reliability of BESS diagnosis.

**Graphical Abstract:**

**Figure:**
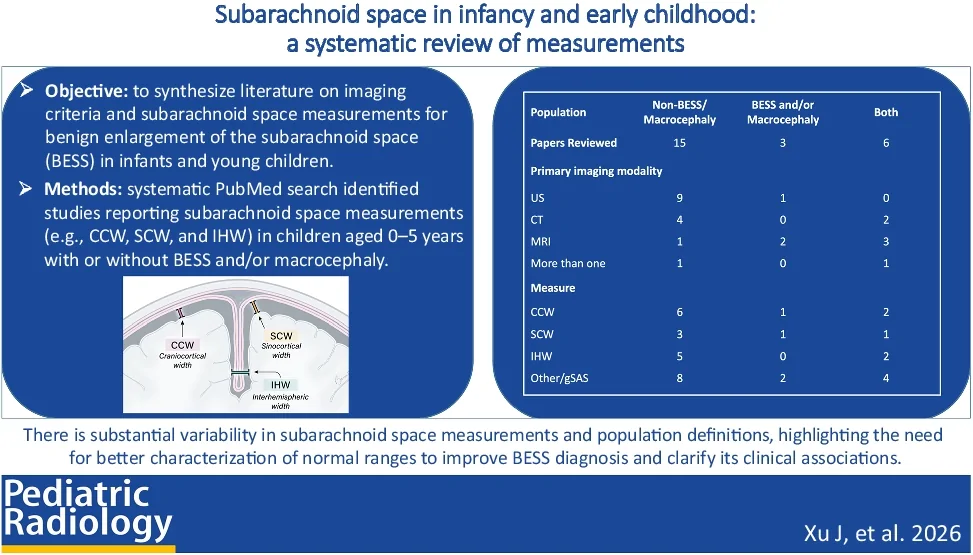

**Supplementary Information:**

The online version contains supplementary material available at https://doi.org/10.1007/s00247-026-06640-z.

## Introduction

Benign enlargement of the subarachnoid space (BESS) is a condition characterized by widened subarachnoid spaces on neuroimaging. BESS is typically associated with macrocephaly or increasing head circumference and enlarged subarachnoid spaces in infancy or early childhood, with stabilization of head growth patterns and resolution of the enlarged subarachnoid space by 2 years of age [1–7]. Literature suggests that BESS has an incidence of approximately 0.4 per 1,000 live births and is more commonly identified in males [8, 9].

BESS is often described as an idiopathic phenomenon [1, 6, 10]. It has been proposed that BESS arises due to delayed maturation of the arachnoid villi causing insufficient absorption of cerebrospinal fluid (CSF), resulting in CSF accumulation and subsequent subarachnoid space enlargement [6, 11–14]. While literature often describes BESS as generally asymptomatic and self-limiting [1, 6, 15], BESS has also been associated with developmental delay by neuropsychiatric testing in more than 30% of subjects [16]. Gross motor delays were the most frequently reported developmental abnormality among subjects with BESS, with most cases demonstrating resolution by approximately 22 months of age [16]. Additionally, the prevalence of autism spectrum disorder in subjects with BESS has been reported to exceed that of the general population [16, 17].

The clinical contextualization of BESS is also relevant when considering its association with subdural collections. Literature is varied with regard to if and what type of subdural collections may be predisposed by BESS, with reported prevalences ranging from 0% to 20% [16, 18–33]. One study reported a prevalence of 42%, in which 7 of 19 subjects with BESS had subdural collections including 3 of 19 (16%) described as hemorrhagic subdural collections [34]. In children under 2 years old, hemorrhagic subdural collections may raise concern for inflicted trauma or physical child abuse including abusive head trauma [30, 35]. Among literature evaluating BESS with subdural collections and additional trauma evaluation, approximately one-third had concomitant injuries concerning for physical abuse; yet, evaluation for additional injuries from suspected abuse was completed less than half of the time [30]. A proposed theoretical model to connect hemorrhagic subdural collections with BESS suggests that the widened subarachnoid space overstretches the bridging blood vessels and makes them more prone to rupture [36]. In contrast, it has also been proposed that BESS may reduce the risk of subdural collections through a dampening effect [37]. Thus, inconsistency in conceptualization of subdural collections associated with BESS represents a key area warranting additional study with clinical relevance.

Establishing clarity and consistency in the diagnosis of BESS is critical for further clinical correlation. Current literature defines BESS using varied diagnostic criteria, methods for measurement, definitions of pathology, and populations. Some studies have characterized the subarachnoid space using subjective grading [3, 32], while others report upper limits to normal subarachnoid space measurements using various measures, such as craniocortical width (CCW), sinocortical width (SCW), and interhemispheric width (IHW) [30]. For example, it has been reported that the upper limits of normal CCW range from 4 mm to 10 mm, the upper limits of normal SCW range from 2 mm to 10 mm, and the upper limits of normal IHW range from 6 mm to 8.5 mm [6].

This lack of consistency in subarachnoid space characterization severely limits the ability to rigorously investigate the clinical trajectories of BESS, including further clarification on proposed association with subdural collections and developmental delays. Given these challenges, we sought to conduct a systematic review and compile a series of studies that quantitatively investigate the subarachnoid space to support delineation of normal versus enlarged subarachnoid spaces, including BESS, across the first years of life. By identifying common themes and techniques across these studies, we aim to provide a clearer understanding of the current state of metrics used to diagnose BESS.

## Methods

We conducted a systematic literature review to capture relevant studies following the Preferred Reporting Items for Systematic Reviews and Meta-Analyses (PRISMA) guidelines [38].

### Exploratory literature review

An exploratory literature review was conducted by one researcher using a manual snowball approach, in which the citations of articles were reviewed and used to identify additional relevant articles to support generation of key terms given known variation in BESS terminology. Starting literature for the snowballing approach focused upon the clinical relevance of subarachnoid space measurements, BESS, and subdural collections in cases of child maltreatment. Start points included seminal articles identified by clinical expertise along with recent topic review [30]. Articles were reviewed if they were referenced by a starting article or cited a starting article and, upon further assessment, described the methodologies used to evaluate the subarachnoid space or BESS in infants or young children. There was focus on identifying articles describing imaging methods and quantitative measurements of the subarachnoid space.

### Literature search

Preliminary findings from the exploratory literature review informed inclusion and exclusion criteria and relevant keywords for the systematic review.

On 12 September 2024, the literature search was conducted in PubMed using the following keywords: ((“subarachnoid space”[MeSH Terms] OR “extraaxial space”[All Fields] OR “benign subarachnoid space”[All Fields] OR “enlarged subarachnoid”[All Fields] OR “external hydrocephalus”[All Fields] OR “benign enlargement”[All Fields] OR “benign macrocephaly”[All Fields] OR “dilated subarachnoid space”[All Fields] OR “enlarged CSF spaces”[All Fields] OR “extra-axial fluid collections”[All Fields] OR “extracerebral”[All Fields]) AND (“subarachnoid”[All Fields] OR “extraaxial”[All Fields])) AND ((english[Filter])) AND (“hasabstract”[All Fields]) AND (“infant, newborn”[MeSH Terms] OR “infant”[MeSH Terms] OR “child, preschool”[MeSH Terms] OR “child”[MeSH Terms]). We confirmed that these criteria included relevant articles identified during exploratory literature review.

### Screening

Screening was conducted in two phases using the Rayyan tool [39]. The first phase consisted of screening titles and abstracts by two independent researchers. Upon resolving any differences, the two independent researchers retrieved the entire text of the screened-in titles and abstracts. Full texts were reviewed and assessed for eligibility, and differences were resolved until the two individuals reached consensus on the articles to review.

Articles were included if they provided novel quantitative measurements of the subarachnoid space in children from birth to 5 years of age using neuroimaging of the head with the modalities of computed tomography (CT), magnetic resonance imaging (MRI), or ultrasound (US). Articles that included children or infants with macrocephaly, neurologically “normal” children or infants, or children or infants with BESS were included. Articles were excluded if they were not published in English or did not provide a detailed description of the subarachnoid space measurement. Articles were also excluded if the study primarily contained BESS and/or macrocephaly subjects with accompanying intracranial pathologies (such as subdural collections, subarachnoid hemorrhages, cerebral atrophy, hydrocephalus, or brain tumor), myelomeningocele, meningitis, history of a brain operation or procedure, craniosynostosis, severe head trauma, or were undergoing chemotherapy.

### Data collection process

Data were extracted from relevant literature identified during the exploratory review and the systematic review by December 10, 2024. Data extracted included subject numbers and ages, conditions of study subjects, study geography, neuroimaging modality utilized (US, CT, MRI), subarachnoid space index measures and measurements (if given), statistics (if provided), phrases and definitions used for BESS, and gender differences. Data extraction was imported into the REDCap electronic data capture tool [40, 41].

### Verification process

Full-text article reviews were manually assigned to one of four board-certified clinicians (three child abuse pediatricians and one pediatric radiologist). Clinicians verified article relevance and extracted data. After clinician review, each article was reassessed, and discrepancies in extracted data were corrected if necessary. Discrepancies and disagreements were resolved through discussion and consensus between researchers and clinicians.

### Data synthesis

During the data extraction process, each article was reviewed for details of their quantitative measures of the subarachnoid space, and numerical measurements were synthesized to facilitate comparison across articles. Studies generally included subjects with either BESS (qualitative or quantitative enlargement of the subarachnoid space), macrocephaly, or both conditions (hereafter “BESS and/or macrocephaly”), subjects without BESS or macrocephaly (hereafter “non-BESS/macrocephaly”), or a combination of both. Some articles provided a total range of linear measurements for the subarachnoid space. Several articles provided means and standard deviations, while other articles provided equations to convey the association of subarachnoid space width and age. To facilitate synthesis, lower and upper edge measurements for the population of interest were extracted if defined by the article. The age range of the subjects was converted to months if necessary (1 week=0.23 months) and corrected for prematurity when relevant data were available.

**Fig. 1:**
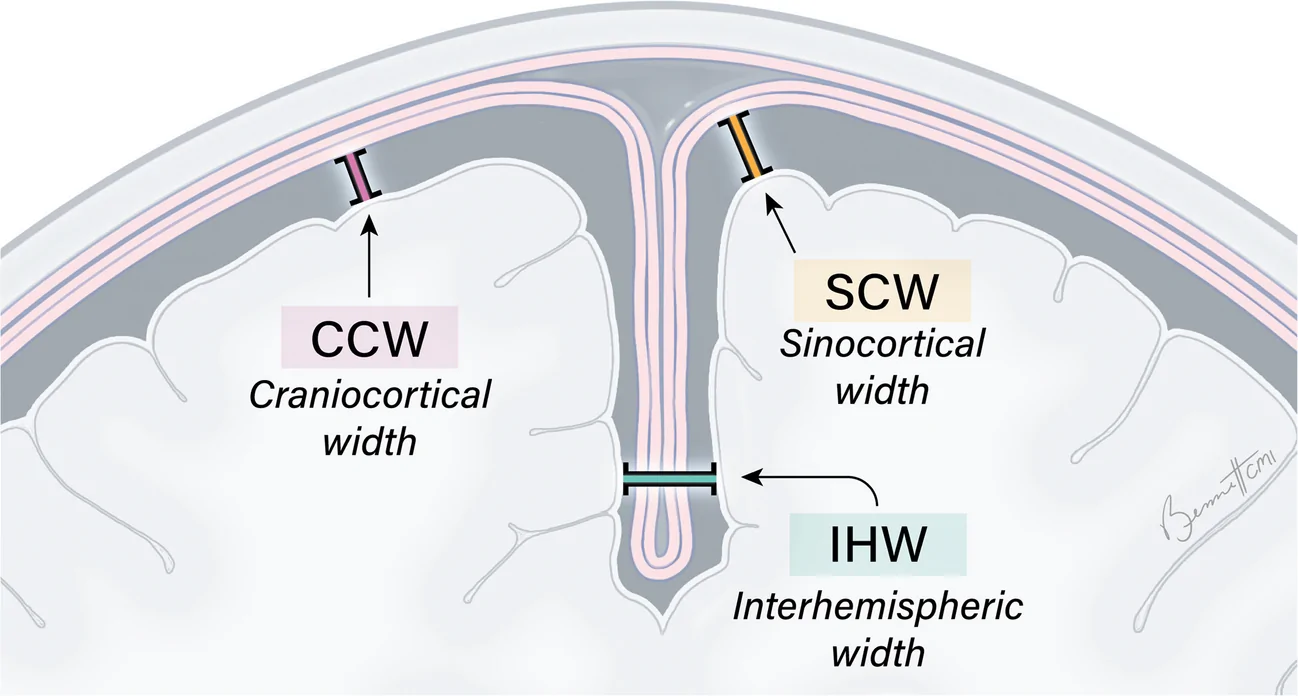
Coronal illustration of craniocortical width (cortex to skull), sinocortical width (cortex to superior sagittal sinus), and interhemispheric width (distance between two cerebral hemispheres)

The common subarachnoid space measures extracted were CCW, SCW, and IHW (Fig. 1). We applied general definitions of CCW to be the distance between the skull and the cortex [42, 43], SCW as the space between the cortex and the overlying superior sagittal sinus [42–44], and IHW as the distance between the two cerebral hemispheres [43]. When literature methods aligned to one of these, measurements were extracted and designated as one of the specified common measures. For example, Hussain et al.’s description of the CSFW as the width from inside the cranium to the gyral apex of the cortex closely aligned with our description of CCW [45]. Measurements of CSFW from this report were subsequently labeled as CCW measurements. Measurements which were not expressly designated or were without detailed descriptions able to be aligned as one of CCW, SCW, or IHW were extracted and reported as general subarachnoid space (gSAS) measurements. For example, Prassopoulos et al. reported measurements for the “width of the frontal subarachnoid space,” which does not provide precision to categorize as one of the above measures, so they were categorized as gSAS measurements [9]. Additional parameters describing measurement methods were extracted as available, including anatomic landmark level.

## Results

### Study identification

Preliminary findings from the exploratory literature review resulted in the identification of twenty-nine potentially relevant studies.

**Fig. 2:**
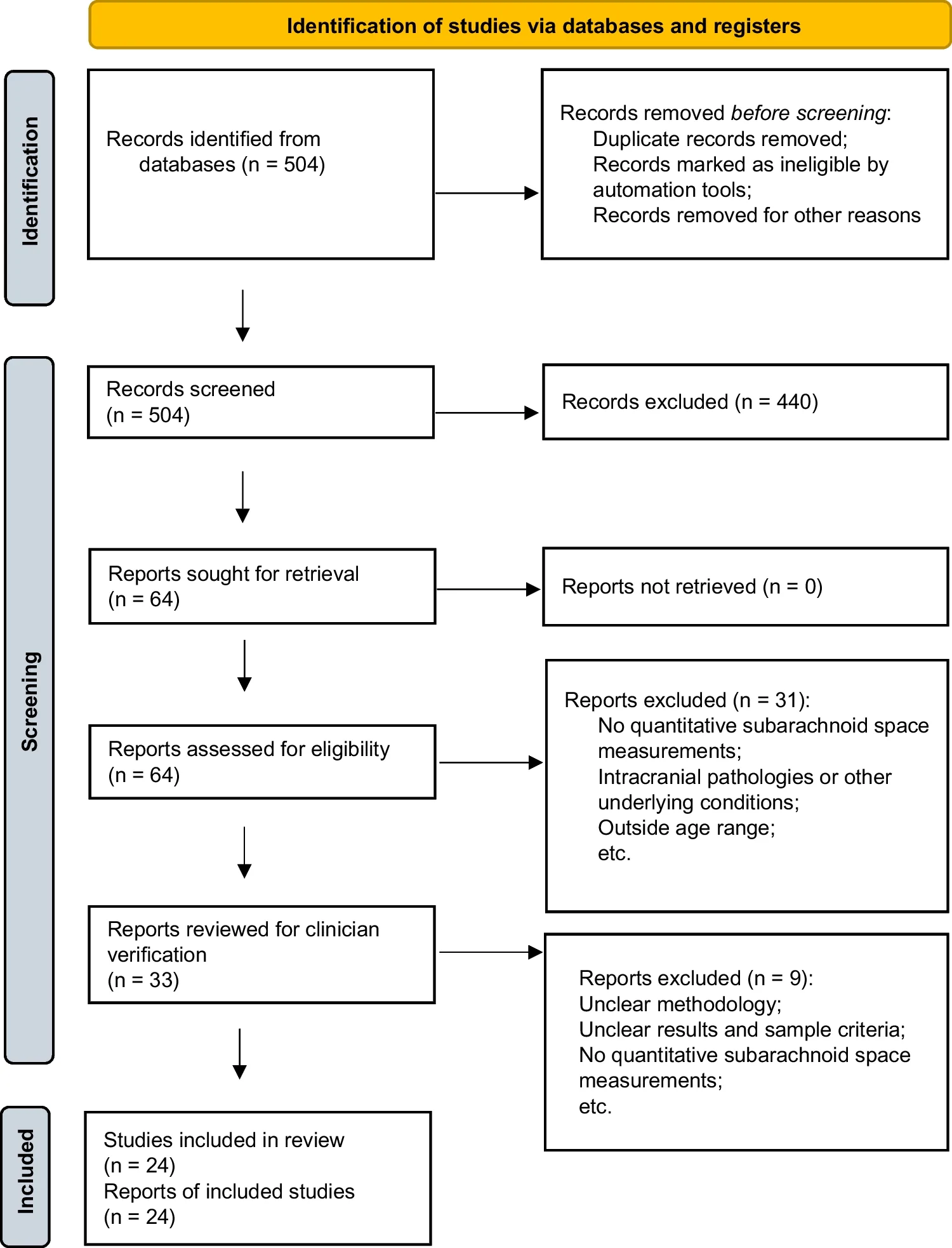
Flow diagram for systematic review of 504 records that were identified during the literature search, with 24 studies included in the final analysis

Using refined keywords, inclusion, and exclusion criteria informed by the preliminary findings, five hundred four records were then identified in the literature search. Twenty-seven of twenty-nine studies from the original exploratory literature review were identified by the refined search criteria, suggesting adequacy of search criteria to identify literature of interest. Of these, four hundred forty were excluded after screening the title and abstract (Fig. 2). Records were screened out due to factors such as lack of quantitative subarachnoid space measurements, scope limited to qualitative descriptions of case studies, focus on the perioptic subarachnoid space, or neurological conditions that affect subarachnoid space anatomy.

Of sixty-four articles included for full-text review, thirty-three articles (including twenty-five from the exploratory literature review) were added to REDCap for clinician verification. Nine articles were subsequently excluded during clinical review. Reasons for exclusion included lack of quantitative subarachnoid space measurements, unclear group distinctions or results, and presence of underlying conditions that could affect brain size or neurological and neurodevelopmental outcomes (e.g., extracranial collections, arachnoidal sylvian cysts, and lipomyelomeningocele). Overall, twenty-four studies were included for the final analysis. Table 1 shows a summary of the characteristics of the twenty-four final studies, and Table 2 describes the individual studies (additional information in Supplementary Material 1). Common neuroimaging modalities were US (*n*=10), followed by MRI (*n*=6), CT (*n*=6), and multiple modalities (*n*=2) (Table 1).

**Table 1:** Summary of 24 studies included in the systematic review

	Population		
	Non-BESS/macrocephaly	BESS and/or macrocephaly	Both
**Number of papers reviewed**	15	3	6
**Publication year range**	1979-2024	1999-2022	1989-2023
**Primary modality**			
** US**	9	1	0
** CT**	4	0	2
** MRI**	1	2	3
** More than one**	1	0	1
**Measure**			
** CCW**	6	1	2
** SCW**	3	1	1
** IHW**	5	0	2
** Other/gSAS**	8	2	4

*BESS*, benign enlargement of the subarachnoid space; *BESS and/or macrocephaly*, either BESS, macrocephaly, or both conditions; *CCW*, craniocortical width; *CT*, computed tomography; *gSAS*, general subarachnoid space measure not expressly designated or aligned with common specified measures (e.g., CCW, SCW); *IHW*, interhemispheric width; *MRI*, magnetic resonance imaging; *non-BESS/macrocephaly*, without BESS or macrocephaly; *SCW*, sinocortical width; *US*, ultrasound

**Table 2:** Description of 24 studies that quantitatively investigate the subarachnoid space to support delineation of normal versus enlarged subarachnoid space, including benign enlargement of the subarachnoid space

Authors	Non-BESS/macrocephaly (*n*)	BESS and/or macrocephaly (*n*)	Corrected age (months)	Modality	Measure	Measure description	Measurements
Fukuyama et al. [46]	6	NA	min: 0 - max: 23	CT	Other/gSAS	“Greatest width of [subarachnoid space] in the slice in which each had the greatest dilatation.”	min: 0 - max: 5.7 mm
Pedersen et al. [47]	46	NA	min: 2.4 - max: 36	CT	IHW	“[Max] width of the interhemispheric fissure.”	5th percentile: 3.3 mm 50th percentile: 3.7 mm 95th percentile: 6.6 mm
Prassopoulos et al. [9]	96	61	min: 3 - max: 27	CT	IHW	“Middle of the anterior part of interhemispheric fissure.”	Non-BESS/macrocephaly: <4 mm BESS: min: 8 - max: 17 mm
					Other/gSAS	“Width of the frontal subarachnoid space.”	Non-BESS/macrocephaly: <4 mm BESS: min: 6 - max: 23 mm
Sawin et al. [48]	20	NA	Mean: 6.4±3.8	CT	Other/gSAS	Undefined	0.6±1.4 ml
Fingarson et al. [49]	66	10	Mean: 6.8±4.65 min: 1 - max: 24	CT	Other/gSAS	Subarachnoid space: “greatest distance from the gyral surface to the inner calvarial cortex.”	2.6±1.5 mm
Yu et al. [7]	538	NA	Mean: 11.15±6.628 min: 1 - max: 24	CT	IHW	“Distance from the medial aspect of one superior frontal gyrus to the medial aspect of the other superior frontal gyrus.”	95th percentile non-BESS/macrocephaly 1 - 12 months: 4.477+0.480age - 0.048age^2 Non-BESS/macrocephaly upper limit for 13 - 24 months: 3.1 mm
					Other/gSAS	CWF: “Distance from the inside of the skull to the superior frontal gyrus.”	95th percentile non-BESS/macrocephaly 1 - 12 months: 3.652+0.700age-0.064age^2 Non-BESS/macrocephaly upper limit for 13 - 24 months: 2.8 mm
Alper et al. [18]	NA	13	Mean: 7.2 min: 3 - max: 17	MRI	Other/gSAS	Undefined	8.0±1.4 mm
Danzer et al. [50]	9	NA	Mean: 0.4±0.12	MRI	Other/gSAS	CSF space at the level of the frontal horns: difference between the linear transverse dimension of the skull and the brain at the level of the frontal horns CSF space at the level of the atria: difference between the linear transverse dimension of the skull and the brain at the level of the atria	Total CSF space at frontal horns: 2.20±0.5 mm Total CSF space at atrium: 2.08±0.4 mm
Hussain et al. [45]	150	10	Non-BESS/macrocephaly: 11.1±7.6 BESS and/or macrocephaly: 10.6±7.8 min: 0 - max: 25	MRI	CCW	CSF width (CSFW): “[max] width of CSF, from inside the cranium to a gyral apex of the cortex.”	Non-BESS/macrocephaly: 4.58±2.25 mm Benign external hydrocephalus: 7.96±4.79 mm
					IHW	“[Max] distance in the frontal interhemispheric fissure between gyri.”	Non-BESS/macrocephaly: 3.89±1.83 mm Benign external hydrocephalus: 6.30±2.79 mm
					Other/gSAS	“[Max] distance from the outside of the skull to a gyral apex of the cortex.”	Non-BESS/macrocephaly mean: 4.58±2.25 mm Largest CCW of a child diagnosed as “normal” was 14.81 mm
Whitehead et al. [51]	21	53	Non-BESS/macrocephaly: 13±6 min: 1 - max: 25 BESS and/or macrocephaly: 13±8 min: 0.3 - max: 34	MRI	CCW	Max “width of the frontal subarachnoid space (from the calvarial inner table to the surface of the superior frontal gyrus of the frontal pole).”	Normocephalic mean: 2.4 mm Macrocephalic mean: 6.4 mm
Alshareef et al. [19]	NA	36	Mean: 10.25±5.8	MRI	Other/gSAS	“Perpendicular distance between the inner table of the calvarium to the cortical surface of the frontal lobes.”	10±4.9 mm
Ho et al. [52]	15	22	Non-BESS/macrocephaly: min: 5 - max: 36 BESS and/or macrocephaly: min: 3 - max: 22	MRI	Other/gSAS	Volume	Non-BESS/macrocephaly mean: 78.1±17.7 ml ESSI mean: 223.2±20.2 ml
Greiner et al. [21]	60	NA	Median: 11.9±4.3 min: 2.1 - max: 23.2	MRI, CT	Other/gSAS	“Largest distance of the frontal [subarachnoid space] perpendicular to the calvaria,” measured from the inner table to the cortex surface	2.8±1.1 mm (min: 1.1 - max: 5.6 mm)
Libicher and Tröger [43]	89	NA	Mean: 3.5 min: 0.03 - max: 12	US	CCW	“Shortest vertical distance between the calvarium and the surface of the cerebral cortex.”	min: 0.3 - max: 6.3 mm
					SCW	“Shortest distance between the lateral wall of the triangular SSS and the surface of the adjacent cerebral cortex.”	min: 0.4 - max: 3.3 mm
					IHW	“Widest horizontal distance between the hemispheres.”	min: 0.5 - max: 8.2 mm
Frankel et al. [53]	82	NA	min: 0 - max: 0.06	US	CCW	“Distance from the inner table of the calvarium to the surface of the immediately subjacent cerebral cortical gyrus and the distance from the wall of the superior sagittal sinus to the cortical surface.”	NA
					SCW	“Shortest distance between the lateral wall of the triangular superior sagittal sinus and the surface of the adjacent cerebral cortex.”	NA
					Other/gSAS	Mean fluid width: CCW and SCW were obtained bilaterally and averaged to obtain a mean fluid width	Mean: 1.57 mm min: 0 - max: 3.3 mm
Fessell et al. [42]	NA	38	Mean: 6.7±4.4 min: 0.7 - max: 20.7	US	CCW	“Shortest distance from the calvarium to the surface of the cerebral cortex.”	*n*=18: 7±1.6 mm (min: 4 - max: 10 mm)
					SCW	“Shortest distance from the lateral wall of the superior sagittal sinus to the surface of the cerebral cortex.”	*n*=20: 7±1.8 mm (min: 3 - max: 10 mm)
Lam et al. [10]	278	NA	Mean 1.8 min: 0.03 - max: 12.5	US	CCW	“Widest vertical distance between calvarium.”	2.32845+0.208036×age-0.003709×age^2
					SCW	“Widest distance between lateral wall of SSS and surface of cortex.”	2.220131+0.12409×age-0.002252×age^2
					IHW	“Widest horizontal distance between the hemispheres.”	2.874066+0.12765×age-0.002084×age^2
Armstrong et al. [54]	201	NA	Mean: 0.15±0.09	US	SCW	“Lateral edge of the [SSS] to the surface of the brain.”	1.56±0.95 mm
Anderson et al. [55]	48	NA	Mean: 0 min: −0.5 - max: 0.5	US	CCW	Not specified	Mean: 2.7 mm min: 0.5 - max: 8.2 mm
Narli et al. [56]	230	NA	min: 0.03 - max: 0.9	US	CCW	“Widest vertical distance between cortex and calvarium.”	1.261±0.784 mm (min: 0.0 - max: 5.0)
					Other/gSAS	Falx-cortex width: “widest horizontal distance between cortex and falx.”	1.227±0.633 mm (min: 0.0 - max: 5.0)
Okur et al. [57]	100	NA	min: 0 - max: 19	US	CCW	“Shortest distance between calvarium and cortex.” Averaging bilateral measurements	Overall subarachnoid space: mean 3.1 mm (min: 0.5 - max: 6 mm) 0 - 6 months (*n*=65): 3.06±1.14 mm 7 - 12 months (*n*=23): 3.4±1.4 mm >13 months (*n*=12): 2.7±1.3 mm
Ng et al. [58]	57	NA	Mean: −1	US	Other/gSAS	Undefined	2.1±0.062 mm
Fandak et al. [59]	359	NA	Median: 1.5 min: 0.03 - max: 19.7	US	CCW	“Shortest vertical width between the inner table of the calvarium and the surface of the subjacent cortical gyrus at the first cortical bulge within 3 cm of the midline falx.” Obtained bilaterally	min: 0.525 - max: 10.866 mm
					IHW	Minimal IHW: “The smallest horizontal width between the surfaces of the most superior cerebral gyri.” Maximal IHW: “The largest horizontal width between the surfaces of the most superior cerebral sulcations.”	Minimal IHW: min: 0 - max: 6.231 mm Maximal IHW: min: 0.606 - max: 10.614 mm
Govaert et al. [44]	9	8	Non-BESS/macrocephaly: min: 0.07 - max: 0.2 BESS and/or macrocephaly: min: 1 - max: 12.5	US, CT	SCW	Shortest distance “between the lateral wall of the triangular superior sagittal sinus and the surface of the adjacent cerebral cortex.”	Non-BESS/macrocephaly: <2 mm BESS and/or macrocephaly: >2 mm

Mean and standard deviation (SD) depicted as mean±SD; range depicted as minimum – maximum; *BESS*, benign enlargement of the subarachnoid space; *BESS and/or macrocephaly*, either BESS, macrocephaly, or both conditions; *CCW*, craniocortical width; *CSF*, cerebrospinal fluid; *CT*, computed tomography; *gSAS*, general subarachnoid space measure not expressly designated or aligned with common specified measures (e.g., CCW, SCW); *IHW*, interhemispheric width; *Max*, maximum; *Min*, minimum; *MRI*, magnetic resonance imaging; *n*, number of subjects; *NA*, not applicable; *Non-BESS/macrocephaly*, without BESS or macrocephaly; *SCW*, sinocortical width; *SSS*, superior sagittal sinus; *US*, ultrasound

### Subarachnoid space measurement methods

One point of variation included specification of measurement level, defined by anatomic landmarks along the inferior-to-superior axis. Fifteen articles reported measurements of the subarachnoid space, including CCW (*n*=5), SCW (*n*=3), and IHW (*n*=3), without level anatomic landmarks specified (Supplementary Material 1). Of those with level specifications by anatomic landmarks, reports included the foramen of Monro (*n*=5) or alternative specified locations (*n*=4), including the cerebral aqueduct, the lateral ventricle, the frontal horns and atria, or the cerebral gyrus. Of the five articles with measurements at the level of the foramen of Monro, CCW was the most common measurement reported (*n*=4), followed by SCW (*n*=2) and IHW (*n*=2) (Supplementary Material 1). Of the studies that completed imaging by US or in combination with US (*n*=11), a majority reported measurements from the coronal plane (*n*=9) (Supplementary Material 1), while among those that completed imaging by CT, MRI, or in combination with either (*n*=14), a majority reported measurements from the axial plane (*n*=8) (Supplementary Material 1 [7, 10, 18, 19, 21, 42–45, 48–57, 59]).

There was also considerable variability in the measures and definitions. The most common measure reported was CCW (*n*=9) followed by IHW (*n*=7) and SCW (*n*=5) (Table 1). The definitions of CCW, SCW, and IHW varied across studies, with some specifying the widest distances between anatomic landmarks such as Lam et al.’s definition for CCW and SCW, while others specified the shortest distance (Table 2) [10, 42, 49]. Of note, many studies (*n*=14) provided gSAS measurements that were not expressly designated or were unable to be definitively aligned to one of three common measures (CCW, SCW, and IHW) (Table 1). Most studies provided linear measurements in millimeters. One study reported volumetric (milliliters) measurements by semi-automatic segmentation [52].

### Study populations

Among the included twenty-four studies, measurements were provided for a total of 2,731 subjects, of which 2,480 were non-BESS/macrocephalic, and 251 were BESS and/or macrocephalic, all ranging in age from 0 months to 36 months (Table 2). Studies with subjects described as exclusively comprising BESS and/or macrocephalic subjects ranged in age from 0.7 months to 20.7 months. Seventeen of twenty-four studies referenced gestational age or prematurity, including ten studies that excluded premature infants (Supplementary Material 1). Two studies originated from the same institution and authors, possibly suggesting some overlap of subjects [42, 53]. Otherwise, subjects were assumed to be independent.

Most (*n*=15) studies included subjects expressly understood to be non-BESS/macrocephalic, six included both non-BESS/macrocephalic subjects as well as BESS and/or macrocephalic subjects, while three included subjects described as exclusively comprising of BESS and/or macrocephalic subjects (Table 1). Within the nine studies that included BESS and/or macrocephalic subjects, criteria to define BESS included a variety of methods that reflected clinical impressions with or without quantitative measurements (Supplementary Material 1). Specifically, clinical impression criteria included review by neuroradiology or terms in patient history of “extraaxial hydrocephalus,” “extra-axial hydrocephalus,” or “external hydrocephalus,” with one report specifying assessment of symmetrical widening of the frontal or frontoparietal CSF space and enlarged chiasmatic cistern, sylvian fissures, and anterior part of the interhemispheric fissure [9, 19, 45, 51]. Three papers defined BESS as a combination with history of macrocephaly (e.g., head circumference percentiles: unspecified, greater than 95th or 98th percentile) and pre-determined criteria of CCW >4 mm, SCW >3 mm, or gSAS >5 mm [18, 42, 52]. One paper defined an enlarged subarachnoid space based on quantitative measurements alone with pre-determined criteria of gSAS >4 mm without requirement for an enlarged or increasing head circumference (Supplementary Material 1) [49].

### Measurements reported

For US measurements, the most consistently reported measure was CCW. For CCW, the upper edge of measurements reported in subjects of non-BESS/macrocephaly had maximum measurements reported by US studies from 5 mm to 10.86 mm (Table 2) [56, 59]. These measurements overlap with CCW US measurements within one article that focused on macrocephaly subjects (4 mm to 10 mm) [42]. Supplementary Material 2 provides a visual synthesis of the reported CCW measurements across studies by age, illustrating variability in ranges, means, and equation-derived estimates. As a visual aid to highlight the heterogeneity in reported measurements, this figure is intended for descriptive purposes only and is presented in the supplementary material. Five US studies reported measurements for SCW, with measurements for BESS and/or macrocephaly subjects ranging from 2 mm to 10 mm and measurements for non-BESS/macrocephaly subjects ranging from 0 mm to 3.5 mm [10, 42–44, 54].

In CT and MRI studies, IHW was the most common measure. Among non-BESS/macrocephaly subjects, IHW measurements reported from CT included upper edge of 3.1 mm to 4.0 mm and 95th percentile measurement of 6.6 mm, while the total range was 8 mm to 17 mm within one study with measurements reported for BESS and/or macrocephaly [7, 9, 47]. IHW measurements reported from an MRI study included an average of 3.89 mm±1.83 among non-BESS/macrocephaly while reporting an average of 6.30 mm±2.79 among BESS and/or macrocephaly subjects (Table 2) [45].

Many measures were gSAS. In general, for each study that included both non-BESS/macrocephaly and BESS and/or macrocephaly subjects, measurements reported were greater among BESS and/or macrocephaly subjects. For example, Prassopoulos et al. reported measurements that were larger for the “width of the frontal subarachnoid space” among BESS subjects with measurements of 6 mm to 23 mm compared with non-BESS subject measurements (<4 mm) (Table 2) [9].

In regard to the age range of 0–36 months, when measurements were reported by age bin or continuously, measurements were reported to increase with age. For example, in two studies that generated equations to model subarachnoid spaces by continuous age between 0 months and 24 months, the slopes of the associations were positive (Table 2) [7, 10].

## Discussion

Our systematic review aimed to summarize quantitative measurement of the subarachnoid space to delineate a range of normal measurements in infancy and early childhood to advance demarcation of BESS. Our systematic review included 24 studies that provided quantitative measurements of the subarachnoid space, mostly from non-BESS/macrocephalic subjects. Notable variation in measurement techniques and results was identified. Our results show broad ranges in reported measurements, including from non-pathologic subjects, as well as variation in population definition. These observations support the need for better understanding of the normal patterns of subarachnoid space measurement in order to improve the diagnosis of BESS. Improved definition of BESS is also needed to clarify proposed clinical associations, such as with subdural collections [16–34], particularly given the potential concerns for inflicted trauma or physical child abuse in settings of hemorrhagic subdural collections [30, 35].

Within studies that included both non-BESS/macrocephaly subjects and BESS and/or macrocephaly subjects, subarachnoid space measurements were generally larger in BESS and/or macrocephaly subjects compared to non-BESS/macrocephaly subjects, affirming the base clinical impression that perceivable differences exist, reinforcing the relevance of BESS as a clinical condition warranting further evaluation [9, 44, 45, 51, 52]. Yet, our review also found variable measurement methods across studies. While we found that studies often used MRI and CCW to assess subarachnoid spaces, there were many points of variation. For example, the definitions of commonly used measures were inconsistently reported. When SCW was first introduced, it was defined as the shortest distance between the cortex and the sagittal sinus [44]. Subsequent literature has contrastingly defined SCW as the widest distance [10]. Similarly, CCW has been described as both the shortest and widest distance from the calvarium to the cortex [8, 10, 42, 56]. IHW may also vary in width at different depths [53]. Additionally, some studies reported measurements as the averaged value between the left and right hemispheres or the average distance from various points on the cortex, while other studies reported a general subarachnoid space measure not expressly designated or aligned with specific measures, such as the total sum combining measurements of the left and right hemispheres [12, 19, 49]. This variability in imaging and measurement methods impacts the ability to compare subarachnoid space measurements across studies, hindering the ability to reach standardized measurement guidelines to distinguish BESS.

Additional factors that may influence delineation of BESS include methods of differentiation between subarachnoid and subdural collections, as well as characterization of ventricular volume. For example, of the twenty-four studies, seven studies described their methodology for differentiating enlarged subarachnoid spaces from subdural collections, a key step in supporting the benign designation of findings [18, 19, 21, 42, 45, 51, 59, 60]. Aspects of this approach included signal intensity relative to cerebrospinal fluid, location of cortical veins, displaced arachnoid membranes, compression of brain tissue, or linear measurements [18, 19, 21, 45, 51]. Ventricular volume may also vary within characterization of BESS [6, 9, 30, 42, 48, 52]. Seven of the nine studies that looked at subjects with BESS and/or macrocephaly considered ventricular volume. Details included excluding patients with substantial ventricular enlargement (*n*=2) or providing linear or volumetric measurements of the lateral or third ventricles (*n*=5) [9, 18, 19, 45, 49, 51, 52].

Additional clinical aspects of BESS delineation are also varied. Among studies focusing on infants and young children presenting with macrocephaly or an increasing head circumference, case-finding criteria are inconsistent [1, 8, 18, 21, 24, 42, 61–63]. Some studies focused on subjects with enlarged head circumferences at or above the 95th percentile on standard growth curves [1, 8, 21, 24, 42, 61, 62], while others focused on head circumference greater than the 98th percentile [18, 63]. Additionally, while BESS is commonly associated with macrocephaly or rapidly increasing head circumference, head growth dynamics associated with BESS and macrocephaly may be different from situations of macrocephaly in isolation, and not all infants or children with macrocephaly have BESS [64]. Moreover, there are several standard growth charts available for head circumference, which have been shown to perform differently in macrocephaly identification in typical care scenarios [65]. For all factors above, BESS studies may be inconsistently reporting macrocephalic subjects.

### Limitations

There are several limitations in this review. The primary limitation of this work is the heterogeneity of study methods used to assess the subarachnoid space. For example, there is no consensus on the specific level at which to measure the subarachnoid space. While some studies measured the subarachnoid space at the level of the foramen of Monro [10, 19, 43, 60], other studies did not specify this information or measured the subarachnoid space per a different referrent (e.g., lateral ventricle) [49]. Differences in the techniques used for CT, US, and MRI studies, such as in slice thickness in CT and MRI studies or MRI sequence selection in MRI studies, can also influence image clarity and the ability to extrapolate measurements between scans, further contributing to variability between study methods. Another limitation of this study may be due to the difficulty of differentiating between fluids in subdural collections and those in the subarachnoid space by early imaging techniques [14, 34, 61, 66, 67]. Consequently, some earlier articles may have included subdural collections in their measurements of the subarachnoid space.

With regard to consistency of measurement, our methods attempted to synthesize reports to support consistency in reported measurement methods across literature. For example, the definition of CCW variably included maximum versus minimum distances. In one instance, the reported CCW value included the skull width; we elected to use a different measurement value reported within the paper given that the description was more in line with our understanding of the most common application of CCW [45]. Additionally, subjects in this review ranged from 0 months to 36 months of age. Our attempt to provide insight into variation with age (Supplementary Material 2) demonstrates the need for further study given variability in results available.

Lastly, our search criteria may have missed relevant literature in two ways. First, our search criteria were developed with a focus on clinical delineation of abnormal or enlargement of the subarachnoid space which may have failed to identify the full cadre of literature available on normal or routine measurements. Second, within literature, there have been many terms used to describe clinical conditions synonymous or closely related to BESS, including benign external hydrocephalus [6, 8, 20, 26, 33, 36, 45, 68], idiopathic external hydrocephalus [24], external hydrocephalus [10, 13, 31, 60, 62, 63, 69–74], benign infantile hydrocephalus [75], benign subarachnoid fluid collections [2], enlargement of the subarachnoid spaces [4, 29, 74, 76, 77], benign extracerebral fluid collection [12, 25, 78], benign extra-axial collections of infancy [3], benign enlargement of the frontal subarachnoid space [9], pericerebral fluid collection [79], extraaxial collections [53], benign macrocrania of infancy [23], enlarged subarachnoid spaces of infancy [52], enlarged subarachnoid space [21, 80], benign enlargement of the subarachnoid spaces of infancy [19], benign expansion of the subarachnoid spaces [16], and arachnoidomegaly [81].

### Future directions

In sum, it is imperative to further standardize measurement guidelines to improve reliability and consistency in BESS diagnosis, assess its clinical implications, and support more granular investigations into the condition. Our review supports further investigation into normative subarachnoid spaces in infancy and early childhood and differentiation relevant to BESS diagnosis. Additional clarity into macrocephaly delineation and the overlap of macrocephaly with BESS is also encouraged. Approaches may include prospective study of a large population of children or secondary use of harmonized MRI scans, as has been completed for brain “growth chart” with controls for age, sex, and technical differences [82]. Beyond two-dimensional measures, volumetric analysis of the cerebrospinal fluid in the subarachnoid space has been demonstrated as an emerging approach [52, 62]. The forthcoming abundance of large-scale datasets and robust statistical benchmarking frameworks, coupled with recent advances in automated quantitative tools for multimodal brain image processing, provide a promising foundation for future development of age-specific standardized diagnostic criteria for BESS.

## Conclusion

This review provides valuable insight into the variability of measurement techniques, characterization, and measurements for the subarachnoid space in the context of BESS diagnosis. This lack of consensus hinders the development of standardized diagnostic criteria and limits the ability to assess its clinical implications reliably. As a next step, we recommend development and implementation of consensus standards of measurement approaches that specify parameters such as relevant anatomic landmarks, plane, and widest versus average of multiple measures. Moreover, additional clarity into the definition of associated macrocephaly in settings concerning for BESS should also be considered. In summary, it is imperative to establish standardized age-specific diagnostic criteria to enhance the accuracy of BESS diagnosis and improve understanding of its clinical relevance.

## Supplementary Information

Below is the link to the electronic supplementary material.

Supplementary file1 (DOCX 334 KB)

## Data Availability

No datasets were generated or analysed during the current study.
