# Supplementary material for: Subarachnoid space in infancy and early childhood: a systematic review of measurements
Source: Pediatr Radiol. 2026 Jun 6;56(11):2549–63. doi: 10.1007/s00247-026-06640-z (PMC13430205; doi:10.1007/s00247-026-06640-z)
Supplement: Supplementary file 1 [file 247_2026_6640_MOESM1_ESM.docx]

# Supplementary Material

## Supplementary Material 1

**Supplementary Material 1** Additional description of 24 studies from Table 2 that quantitatively investigate the subarachnoid space to support delineation of normal versus enlarged subarachnoid space, including benign enlargement of the subarachnoid space

| **Authors** | **Geography** | **BESS and/or**  **Macrocephaly criteria** | **Plane** | **Level** | **Measurement methods** | **Exclusion criteria used in study** | **Premature Subjects** |
| --- | --- | --- | --- | --- | --- | --- | --- |
| Fukuyama et al. 1979 [1] | Japan | NA | NA | No level specified | NA | Subjects with head trauma that did not fit Araki's type I classification and subjects with convulsions, except for "simple febrile seizures, infrequent grand mal attacks or pure petit mal." | Not specified |
| Pedersen et al. 1979 [2] | Germany | NA | NA | No level specified | NA | Premature children and children with "perinatal asphyxia, cerebral concussion, malnutrition, malignant neoplasia and treatment with cytostatic drugs." | Excluded |
| Prassopoulos et al. 1995 [3] | Greece | "Qualitative symmetrical widening of the frontal or frontoparietal CSF space, and enlargement of chiasmatic cistern, sylvian fissures, and the anterior part of the interhemispheric fissure." | NA | No level specified | NA | "Children with genetic syndromes, prematurity, a history of intraventricular or subarachnoid haemorrhage, old head trauma, central nervous system infection [as well as those with] CT findings of atrophy ... or with conditions that may cause brain atrophy (perinatal asphyxia, malnutrition, administration of adrenocorticotropic hormone, steroids, or chemotherapeutic agents)." | Excluded |
| Sawin et al. 1996 [4] | United States | NA | Axial | No level specified | NA | "Children with multiple cranial suture abnormalities, impaired neurological function, developmental delay, concurrent craniofacial anomalies, or incomplete radiographic data." | Not specified |
| Fingarson et al. 2017 [5] | United States | gSAS >4 mm | Axial | Level of lateral ventricle | "Extraaxial space was measured on axial CT scans at the level of the lateral ventricle and averaged between the left and right sides." | NA | Included |
| Yu et al. 2021 [6] | China | NA | Axial | Nearest level of cerebral gyrus |  | "Infants with cerebral focal lesions, prematurity, evidence of increased intracranial pressure (e.g., full bregma), abnormal head circumference, central nervous system infections, trauma, chronic use of steroids or chemotherapeutic agents, history of perinatal asphyxia, and more than mild ventricular and basal cistern dilatation." "Newborns were excluded given the difficulty in determining the extent of neurologic disease using clinical findings." | Excluded |
| Alper et al. 1999 [7] | Turkey | Macrocephaly and enlarged subarachnoid space. Macrocephaly: "head circumference greater than 98th percentile on growth charts on first visit."  Enlargement of the subarachnoid space: CCW > 4 mm, only single fluid layer in pericerebral collection same intensity characteristics of CSF. | Axial and coronal | No level specified | NA | NA | Excluded |
| Danzer et al. 2007 [8] | United States | NA | Axial | Level of frontal horns and atria | T2 weighted MRI | NA | Not specified |
| Hussain et al. 2017 [9] | United Kingdom | Subjects with report of "extraaxial hydrocephalus," "extra-axial hydrocephalus," or "external hydrocephalus," "differentiated from subdural hygromas and collections by observing a continuous CSF intensity without visualization of any membranes." | Axial | No level specified | "All measurements were taken at three times on the right, which were then repeated on the left, before a mean value was determined." | "History or scan indication for condition that has been reported to cause an altered frontal subarachnoid space [(e.g.] intraventricular or subarachnoid haemorrhage, prematurity, genetic syndromes including mitochondrial disorders, meningitis, steroid therapy, chemotherapy,  neurosurgery, trauma, subdural collections, cerebral atrophy)." | Excluded |
| Whitehead et al. 2017 [10] | United States | MRI impression containing "macrocephaly" with enlargement of subarachnoid space of infancy interpreted by neuroradiologists. Subjects with history of imaging related to macrocephaly but then identified as "normal" were excluded. | Axial | No level specified | NA | "Patients with non-communicating hydrocephalus, moderate or greater ventriculomegaly, atrophy, structural bone and/parenchymal abnormalities, abnormal brain signal, hemorrhages, meningitis, and normal imaging." | Included |
| Alshareef et al. 2022 [11] | United States | Evaluated for macrocephaly and had a diagnosis of BESSI by neuroradiology impression. | Coronal | Foramen of Monro |  | "Twenty-one patients were excluded due to no evidence of BESSI on the MRI. Two patients were excluded due to previous diagnosis of neonatal intraventricular hemorrhage. Twelve patients were excluded due to previously known intracranial pathologies including ruptured aneurysms, neoplasm, and congenital brain malformations. Five patients were excluded due to a previous or new diagnosis of hydrocephalus ... Four patients were excluded due to a history of traumatic brain injury at the time of or prior to imaging" | Included |
| Ho et al. 2023 [12] | United States | Referral for macrocephaly and gSAS ≥ 5 mm over frontal convexities | Axial | Level of cerebral aqueduct | NA | "Exclusion for both groups included (1) abnormal study for reasons other than enlarged subarachnoid spaces, including large subdural fluid collections, and (2) genetic or syndromic pathology from the electronic medical record." | Not specified |
| Greiner et al. 2013 [13] | United States | NA | Axial | No level specified | NA | Children over 24 months of age, subjects with technically nondiagnostic examinations, "brain tumors, prior intracranial surgery, shunts, craniosynostosis, prior intracranial hemorrhage, prior meningitis/abscess, or parenchymal abnormalities on imaging." "Patients with ventricular enlargement out of proportion to cerebral sulcal prominence suggesting the possibility of hydrocephalus." Children with history of "chemotherapy, brain irradiation, failure to thrive or malnutrition, chronic systemic illness, chronic corticosteroid therapy, significant prematurity (GA 28 weeks), congenital anomalies, or brain atrophy pattern by imaging." | Not specified |
| Libicher & Tröger 1992 [14] | Germany | NA | Coronal | Foramen of Monro | Measured on both sides of the hemispheres | "Children with conditions that might alter the subarachnoid space (i.e., any disease or alteration of the CNS, delayed psychomotor development, dehydration, or prematurity)." Infants with head circumference, body length, and body weight outside of the third and 97th percentile. | Excluded |
| Frankel et al. 1998 [15] | United States | NA | Coronal and sagittal | No level specified | NA | Children with "any neurologic abnormality requiring additional or nonroutine care ... detected on a subsequent well-child visit." | Included |
| Fessell et al. 2000 [16] | United States | Clinical history of enlarged head size (occipital frontal circumference ≥ 95th percentile), CCW >4 mm, and SCW >3 mm. | Coronal | No level specified | "The largest extraaxial fluid collection noted in any coronal plane was measured to determine the CCW and SCW." | Children with a reported neurologic abnormality (i.e., developmental delay such as language and gross motor development delays) or additional sonographic abnormalities. | Not specified |
| Lam et al. 2001 [17] | Hong Kong | NA | Coronal | Foramen of Monro | Measurements were performed on either side of the cerebral hemispheres (cited Libicher and Tröger 1992 as saying the subarachnoid space was symmetrical, and size difference in most measurements was less than 1 mm). "The measurements were performed twice per child and were averaged." | Children with underlying diseases such as "vomiting, sepsis, neoplasm, failure to thrive, chemotherapy, or renal failure." | Excluded |
| Armstrong et al. 2002 [18] | New Zealand | NA | Coronal | No level specified | "There was no significant difference in the width of the subarachnoid space over the right and left cerebral hemispheres (mean difference, 0.6 mm; p = 0.16), [so] a mean width of the subarachnoid space was calculated for each study." | Three scans from babies treated with corticosteroids after birth were excluded. | Included |
| Anderson et al. 2004 [19] | New Zealand | NA | Coronal and sagittal | No level specified | NA | "Of the 100 infants, ... 34 [were excluded] because the 2-D sonogram and 3-D MRI were performed more than 24 days apart and, in 3, only hard copy images of the 2-D sonogram were available." | Included |
| Narli et al. 2006 [20] | Turkey | NA | Coronal | No level specified | NA | "Infants with infectious diseases, congenital anomalies and those small for gestational age." | Excluded |
| Okur et al. 2013 [21] | Turkey | NA | Coronal | Foramen of Monro | NA | "Children with any disease (e.g., gastroenteritis, macrocephaly, prematurity) that may affect measurement of head circumference or subarachnoid distance and cases in which US evaluation revealed disorder." | Excluded |
| Ng et al. 2013 [22] | United Kingdom | NA | NA | No level specified | NA | "Infants born to mothers with known thyroid disease, on antithyroid medications or amiodarone; infants with major congenital or chromosomal abnormalities known to affect thyroid function or brain development; infants whose mothers died within 5 days of giving birth." | Included |
| Fandak et al. 2024 [23] | Switzerland | NA | Coronal | Foramen of Monro | NA | Excluded subjects with "prematurity (born before 37 weeks of gestation), oxygen deprivation at birth, neonatal intensive care unit admission, a history of being small for gestational age, a diagnosed genetic disease, ... [and children that] had previously undergone US, MRI, or CT of the brain or spine as it may indicate neurological pathology." | Excluded |
| Govaert et al. 1989 [24] | Belgium | gSAS cutoff limit >2 mm | Coronal | No level specified | NA | NA | Not specified |

BESS: Benign enlargement of the subarachnoid space; BESS and/or macrocephaly: Either BESS, macrocephaly, or both conditions; BESSI: Benign enlargement of the subarachnoid spaces of infancy; CCW: Craniocortical width; CSF: Cerebrospinal fluid; CT: Computed tomography; GA: Gestational age; gSAS: General subarachnoid space measure not expressly designated or aligned with common specified measures (e.g., CCW, SCW); IHW: Interhemispheric width; Level: Level of anatomic landmarks during measurement of subarachnoid space; MRI: Magnetic resonance imaging; NA: Not applicable; Plane: Orientation during neuroimaging; SCW: Sinocortical width; US: Ultrasound

## Supplementary Material 2

**Supplementary Material 2** Reported craniocortical width (CCW) values across non-BESS/macrocephaly cohorts by age (months), summarized from included studies. Each study is represented using a distinct color. Rectangular shaded areas indicate reported ranges (minimum to maximum CCW) across the specified age interval, with dashed horizontal lines marking the upper and lower bounds. Solid horizontal lines represent mean CCW values when reported without standard deviation. When mean and standard deviation were provided, the solid line denotes the mean and the shaded rectangle area spans twice the given standard deviation. Dotted lines indicate values derived from equations reported in the original studies.

Mean, as represented by an equation

Mean, with twice the standard deviation (if available) surrounding the mean

Lower or upper limits of range with corresponding area shaded in between

1.	Fukuyama Y, Miyao M, Ishizu T, Maruyama H (1979) Developmental changes in normal cranial measurements by computed tomography. Dev Med Child Neurol 21:425-432. <https://doi.org/10.1111/j.1469-8749.1979.tb01645.x>.

2.	Pedersen H, Gyldensted M, Gyldensted C (1979) Measurement of the normal ventricular system and supratentorial subarachnoid space in children with computed tomography. Neuroradiology 17:231-237. <https://doi.org/10.1007/bf00337531>.

3.	Prassopoulos P, Cavouras D, Golfinopoulos S, Nezi M (1995) The size of the intra- and extraventricular cerebrospinal fluid compartments in children with idiopathic benign widening of the frontal subarachnoid space. Neuroradiology 37:418-421. <https://doi.org/10.1007/bf00588027>.

4.	Sawin PD, Muhonen MG, Menezes AH (1996) Quantitative analysis of cerebrospinal fluid spaces in children with occipital plagiocephaly. J Neurosurg 85:428-434. <https://doi.org/10.3171/jns.1996.85.3.0428>.

5.	Fingarson AK, Ryan ME, McLone SG, Bregman C, Flaherty EG (2017) Enlarged subarachnoid spaces and intracranial hemorrhage in children with accidental head trauma. Journal of Neurosurgery: Pediatrics PED 19:254-258. <https://doi.org/10.3171/2016.8.Peds16146>.

6.	Yu K, Yi M, Cui R, Gong T, Dong C, Gao X, Zhao J, Li M (2021) Computed Tomography Measurement and Evaluation of the Subarachnoid Space Over Cerebral Convexities in Infants Aged 1-24 Months. J Child Neurol 36:447-452. <https://doi.org/10.1177/0883073820977998>.

7.	Alper G, Ekinci G, Yilmaz Y, Arikan C, Telyar G, Erzen C (1999) Magnetic resonance imaging characteristics of benign macrocephaly in children. J Child Neurol 14:678-682. <https://doi.org/10.1177/088307389901401010>.

8.	Danzer E, Johnson MP, Bebbington M, Simon EM, Wilson RD, Bilaniuk LT, Sutton LN, Adzick NS (2007) Fetal head biometry assessed by fetal magnetic resonance imaging following in utero myelomeningocele repair. Fetal Diagn Ther 22:1-6. <https://doi.org/10.1159/000095833>.

9.	Hussain ZB, Hussain AB, Mitchell P (2017) Extra-axial cerebrospinal fluid spaces in children with benign external hydrocephalus: A case-control study. Neuroradiol J 30:410-417. <https://doi.org/10.1177/1971400917719298>.

10.	Whitehead MT, Lee B, McCarron A, Fricke ST, Vezina G (2017) Reduced subarachnoid fluid diffusion in enlarged subarachnoid spaces of infancy. Neuroradiol J 30:418-424. <https://doi.org/10.1177/1971400916689803>.

11.	Alshareef M, Tyler M, Litts C, Pearce J, Yazdani M, Eskandari R (2022) Prevalence of Visible Subdural Spaces in Benign Enlargement of Subarachnoid Spaces in Infancy: A Retrospective Analysis Utilizing Magnetic Resonance Imaging. World Neurosurg 164:e973-e979. <https://doi.org/10.1016/j.wneu.2022.05.079>.

12.	Ho CY, Sankar M, Persohn S, Kralik SF, Graner B, Territo PR (2023) Quantitative assessment of cerebrospinal fluid flow and volume in enlargement of the subarachnoid spaces of infancy using MRI. Pediatr Radiol. <https://doi.org/10.1007/s00247-023-05659-w>.

13.	Greiner MV, Richards TJ, Care MM, Leach JL (2013) Prevalence of subdural collections in children with macrocrania. AJNR Am J Neuroradiol 34:2373-2378. <https://doi.org/10.3174/ajnr.A3588>.

14.	Libicher M, Tröger J (1992) US measurement of the subarachnoid space in infants: normal values. Radiology 184:749-751. <https://doi.org/10.1148/radiology.184.3.1509061>.

15.	Frankel DA, Fessell DP, Wolfson WP (1998) High resolution sonographic determination of the normal dimensions of the intracranial extraaxial compartment in the newborn infant. J Ultrasound Med 17:411-415; quiz 417-418. <https://doi.org/10.7863/jum.1998.17.7.411>.

16.	Fessell DP, Frankel DA, Wolfson WP (2000) Sonography of extraaxial fluid in neurologically normal infants with head circumference greater than or equal to the 95th percentile for age. J Ultrasound Med 19:443-447. <https://doi.org/10.7863/jum.2000.19.7.443>.

17.	Lam WWM, Ai VHG, Wong V, Leong LLY (2001) Ultrasonographic measurement of subarachnoid space in normal infants and children. Pediatric Neurology 25:380-384. <https://doi.org/10.1016/S0887-8994(01)00349-6>.

18.	Armstrong DL, Bagnall C, Harding JE, Teele RL (2002) Measurement of the subarachnoid space by ultrasound in preterm infants. Arch Dis Child Fetal Neonatal Ed 86:F124-126. <https://doi.org/10.1136/fn.86.2.f124>.

19.	Anderson NG, Warfield SK, Wells S, Spencer C, Balasingham A, Volpe JJ, Inder TE (2004) A limited range of measures of 2-D ultrasound correlate with 3-D MRI cerebral volumes in the premature infant at term. Ultrasound Med Biol 30:11-18. <https://doi.org/10.1016/j.ultrasmedbio.2003.10.001>.

20.	Narli N, Soyupak S, Yildizdaş HY, Tutak E, Ozcan K, Sertdemir Y, Satar M (2006) Ultrasonographic measurement of subarachnoid space in normal term newborns. Eur J Radiol 58:110-112. <https://doi.org/10.1016/j.ejrad.2005.11.007>.

21.	Okur A, Kucuk O, Karacavus S, Yildirim A, Erkoc MF, Erdogan Y, Serin HI (2013) A novel index in healthy infants and children - subarachnoid space: ventricle ratio. Folia Morphol (Warsz) 72:142-146. <https://doi.org/10.5603/fm.2013.0024>.

22.	Ng SM, Turner MA, Gamble C, Didi M, Victor S, Manning D, Settle P, Gupta R, Newland P, Weindling AM (2013) An explanatory randomised placebo controlled trial of levothyroxine supplementation for babies born <28 weeks' gestation: results of the TIPIT trial. Trials 14:211. <https://doi.org/10.1186/1745-6215-14-211>.

23.	Fandak J, Markart S, Willems EP, Wildermuth S, Frauenfelder T, Fischer T, Dietrich TJ, Waelti SL (2024) Prospective measurement of the width of cerebrospinal fluid spaces by cranial ultrasound in neurologically healthy children aged 0-19 months. BMC Pediatr 24:315. <https://doi.org/10.1186/s12887-024-04797-w>.

24.	Govaert P, Pauwels W, Vanhaesebrouck P, De Praeter C, Afschrift M (1989) Ultrasound measurement of the subarachnoid space in infants. Eur J Pediatr 148:412-413. <https://doi.org/10.1007/bf00595899>.
